# Advances in the pharmaceutical treatment options for canine osteoarthritis

**DOI:** 10.1111/jsap.13495

**Published:** 2022-03-14

**Authors:** C. Pye, N. Bruniges, M. Peffers, E. Comerford

**Affiliations:** ^1^ Institute of Life Course and Medical Sciences, Faculty of Health and Life Sciences University of Liverpool William Henry Duncan Building, 6 West Derby Street Liverpool L7 8TX UK; ^2^ University of Liverpool Small Animal Teaching Hospital University of Liverpool Leahurst Campus, Chester High Road Neston CH64 7TE UK

## Abstract

Canine osteoarthritis is a significant cause of pain in many dogs and can therefore compromise animal welfare. As the understanding of the biology and pain mechanisms underpinning osteoarthritis grows, so do the number of treatments available to manage it. Over the last decade, there have been a number of advances in the pharmaceutical treatment options available for dogs with osteoarthritis, as well as an increasing number of clinical trials investigating the efficacy of pre‐existing treatments. This review aims to examine the current evidence behind pharmaceutical treatment options for canine osteoarthritis, including non‐steroidal anti‐inflammatory drugs, piprants, monoclonal antibodies, adjunctive analgesics, structure modifying osteoarthritis drugs and regenerative therapies.

## INTRODUCTION

Osteoarthritis (OA) is a significant cause of pain, lameness and morbidity in dogs and many other species, including humans, across the world (Brown *et al*. 2013b, Knazovicky *et al*. 2016, Anderson *et al*. 2018, Cui *et al*. 2020). It is a multi‐factorial, progressive, degenerative disease of synovial joints, affecting not only the articular cartilage but also other structures within the specific synovial joint (Loeser *et al*. 2012). Degradation of articular cartilage, subchondral bone sclerosis, osteophytosis, varying degrees of synovitis, meniscal and ligament degeneration are all characteristics of the disease process. However, there is still much to be understood regarding the underlying pathogenesis of OA (Glyn‐Jones *et al*. 2015).

Canine OA most commonly arises as a result of inciting factors, such as coxofemoral (hip) joint dysplasia, elbow dysplasia, cranial cruciate ligament (CCL) disease, patella luxation, limb malformations and articular fractures (Johnston 1997). A number of risk factors for canine OA have been identified, including genetic predispositions, diet and obesity, all of which can play a role in disease progression (Anderson *et al*. 2018).

Estimates of the prevalence of dogs presenting with OA to primary care veterinary practices in the UK vary depending on the study, but have recently been reported to be between 2.5% (Anderson *et al*. 2018) and 6.6% (O'Neill *et al*. 2014b). A previous study of dogs attending referral hospitals in the USA reported an estimated prevalence of OA of up to 20% in dogs over 1‐year old (Johnston 1997). The true prevalence of the disease, however, is likely to be higher when unreported cases and the discrepancies in recording systems are taken into consideration (O'Neill *et al*. 2014a). With an estimated UK canine population of around 12.5 million (Pet Food Manufacturers Association 2021) and 77 million in the USA (American Veterinary Medical Association 2017), this represents a significant number of affected dogs, as well as a substantial number of dog owners and caregivers charged with the responsibility (and economic cost) of managing their treatment (Belshaw *et al*. 2020).

As OA, therefore, presents a welfare problem for many dogs, it is important that information regarding treatment options for these animals is both up to date and evidence based, aiding veterinary practitioners and dog owners in effectively managing these cases. With a plethora of treatment options available, and novel drugs having gained market authorisation for the treatment of canine OA in the last decade, the aim of this review is to examine the recent evidence in the literature underpinning the pharmaceutical treatment options for the management of canine OA. In this review, pharmaceutical treatments of canine OA will be discussed, focussing on novel therapies and updated evidence for existing treatments.

Some of the studies referenced in this review compare pharmaceutical treatments of OA to a placebo. It should be noted that using a placebo as a control in a disease that is known to be painful and for which there are licensed treatments available has ethical implications. In the UK, these would, as a minimum, require an Animal Test Certificate from the Veterinary Medicines Directorate or may require authorisation under the Animal (Scientific Procedures) Act 1986 depending on the nature of the study (Veterinary Medicine Directorate 2018). As an alternative to placebo‐controlled trials, some studies use a medication with known efficacy as a positive control to compare to the treatment group (Reymond *et al*. 2012). In these cases, the term “non‐inferior” is used if the treatment group is not worse than the positive control (Freise *et al*. 2013).

Licensing guidelines for the UK market will be used as standard unless stated otherwise. Veterinary practitioners should also be aware of non‐pharmaceutical therapies in the non‐surgical management of canine OA, such as weight management and physiotherapy, enabling a multi‐modal treatment approach to this disease.

## PAIN MECHANISMS IN OA

**FIG 1:**
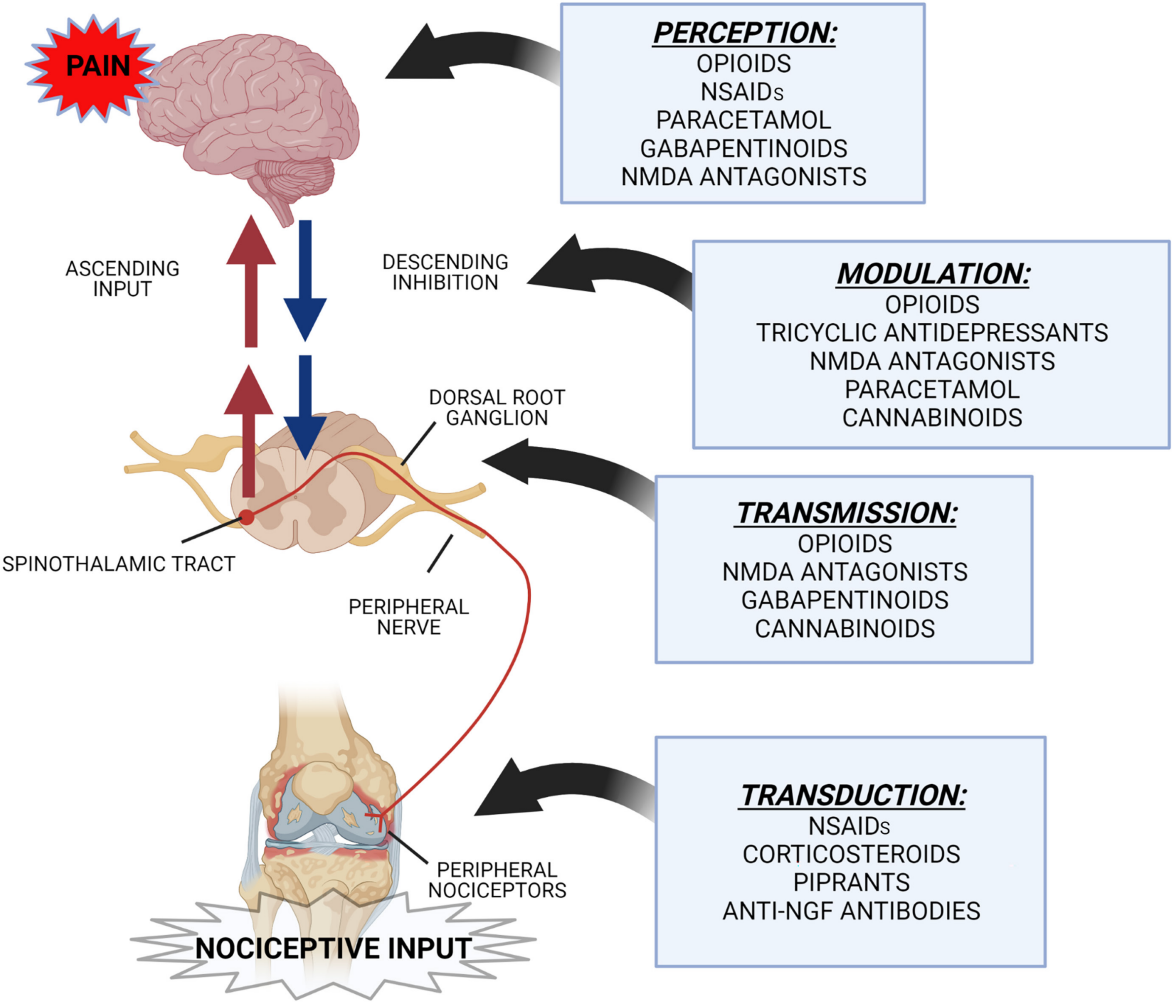
Illustration of an osteoarthritic knee joint showing the four main stages of the pain pathway in osteoarthritis and the targets of certain drugs. Transduction is the conversion of a nociceptive stimulus into electrical impulse. Transmission is the electrical impulse transmitted from peripheral sensory nerves to the central nervous system (CNS). Modulation is how the nociceptive stimulus is processed by the CNS and includes the endogenous opioid system, as well as ascending input and descending inhibitory pathways. Perception is how the brain (particularly the somatosensory cortex) interprets nociceptive inputs resulting in conscious perception of pain. Adapted from Yamaoka & Auckburally (2014)

OA is a painful chronic disease. The pain mechanisms involved are complex (Fu *et al*. 2018). Both peripheral components of pain and central processes are involved, with nociceptive, inflammatory and neuropathic types of pain occurring to varying degrees (White & Hunt 2019). There are several classes of analgesic medications available, with different mechanisms of action, targeting nociception at different steps along the pain pathway (Fig 1). With this in mind, it is important to consider multi‐modal analgesia and management when treating canine OA patients if an insufficient response to one type of medication is shown (Lascelles *et al*. 2008).

## PHARMACEUTICAL TREATMENTS OF CANINE OA

### Non‐steroidal anti‐inflammatory drugs

**FIG 2:**
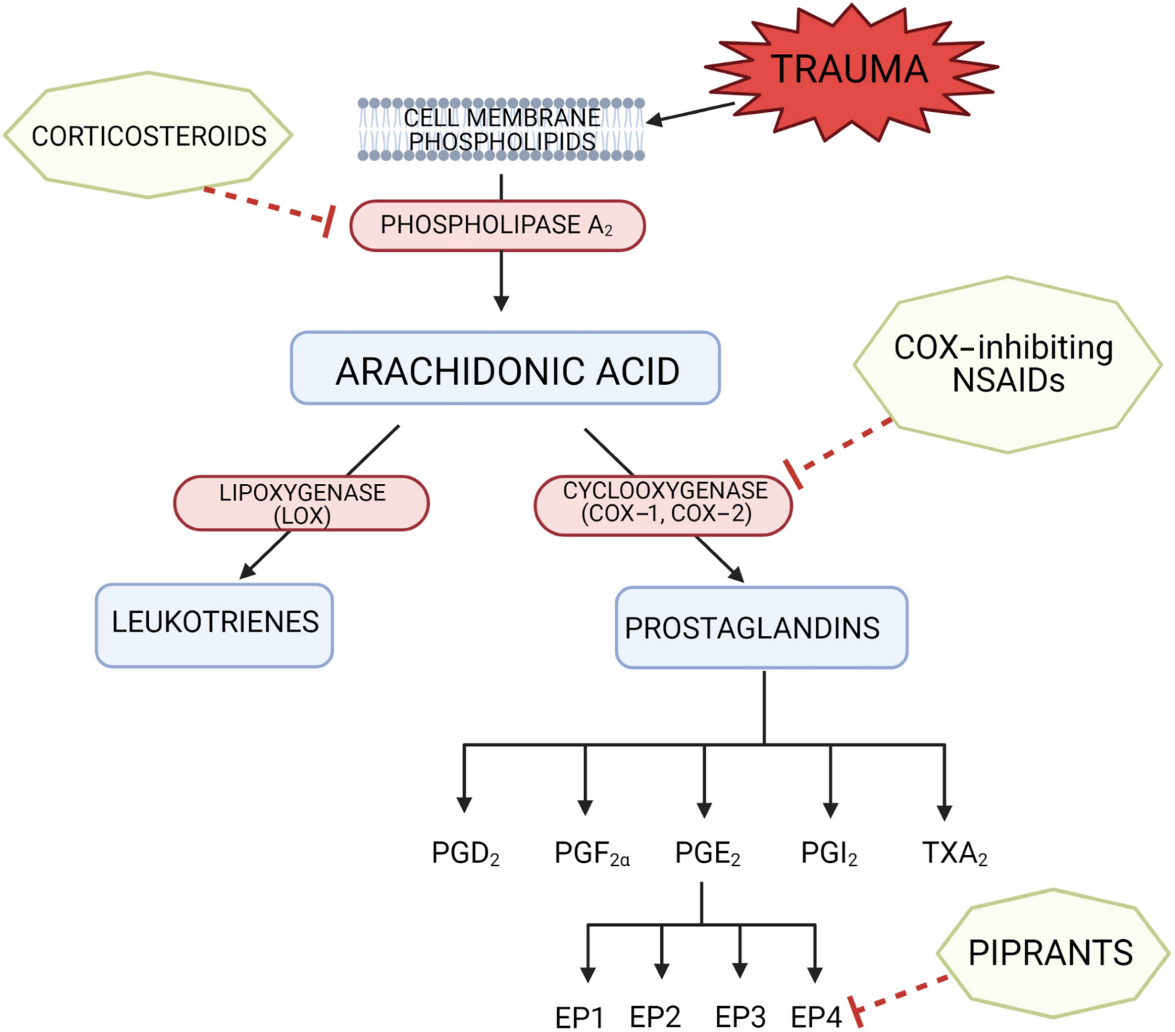
Targets of corticosteroids, COX‐inhibiting NSAIDs and piprants on the pathway of arachidonic acid metabolism. Corticosteroids inhibit phospholipase A2, preventing the conversion of phospholipids to arachidonic acid. NSAIDs inhibit cyclooxygenase, therefore preventing the production of prostaglandins from arachidonic acid. Piprants selectively antagonise the prostaglandin‐E_2_ EP4 receptor. Adapted from Monteiro & Steagall (2019)

Non‐steroidal anti‐inflammatory drugs (NSAIDs) have been first‐line analgesics for the management of canine OA pain for many years. Conventional NSAIDs exert their analgesic effects by inhibiting the enzyme cyclooxygenase (COX), which is responsible for the production of prostaglandins from arachidonic acid. There are two peripheral isoforms of COX: COX‐1 and COX‐2. Unwanted side effects caused by the inhibition of COX‐1, such as gastrointestinal and renal effects, have led to the development of preferential and selective COX‐2 inhibitors (Kukanich *et al*. 2012). The mechanism of action of NSAIDs is outlined in Fig 2.

The efficacy of NSAIDs in reducing pain related to OA has been well documented. Of the NSAIDs licensed for veterinary use, previous systematic reviews of treatments for canine OA have shown a high level of evidence in the literature for the efficacy of meloxicam, carprofen and firocoxib (Aragon *et al*. 2007, Sandersoln *et al*. 2009). Since the publication of these systematic reviews, four other coxibs have been licensed in the UK for the treatment of OA in dogs, namely robenacoxib (Onsior; Elanco), mavacoxib (Trocoxil; Zoetis), cimicoxib (Cimalgex; Vetoquinol) and enflicoxib (Daxocox; Animalcare Limited). Similar to firocoxib, these NSAIDs are highly selective inhibitors of COX‐2, therefore theoretically reducing the risk of adverse side effects caused by COX‐1 inhibition (Toutain *et al*. 2018). In humans, coxibs have been shown to increase the risk of myocardial infarction, leading to some products losing their market authorisation (Arora *et al*. 2020). However, this has not been to date demonstrated in dogs. Due to a lack of sufficiently powered, randomised, blinded, placebo‐controlled studies when comparing the incidence of adverse effects and analgesic efficacy of different NSAIDs, it is not known whether selective COX‐2 inhibitors are superior to preferential COX‐2 inhibitors (Monteiro‐Steagall *et al*. 2013).

A summary of recent clinical trials involving NSAIDs licensed for use in canine OA is shown in Table 1. One study demonstrated a similar efficacy in reducing pain related to OA with robenacoxib compared to carprofen over a 12‐week treatment period in 188 dogs (Reymond *et al*. 2012). Clinical trials involving cimicoxib for the treatment of OA in dogs are lacking. However, one study showed cimicoxib to provide analgesia superior to tramadol postoperatively after tibial plateau levelling osteotomy surgery (Piras *et al*. 2021). Enflicoxib, the most recently licensed of the coxibs, is dosed at weekly intervals, with an initial loading dose of 8 mg/kg, followed by once weekly doses of 4 mg/kg (National Office of Animal Health 2021a). A recent study involving 242 dogs randomised to receive either enflicoxib, mavacoxib or placebo, found an improvement in veterinary and owner assessment of clinical signs related to OA with both NSAIDs compared to placebo over the 6‐week trial period (Salichs *et al*. 2021). Mavacoxib differs from the other licensed NSAIDs, as it has a much longer half‐life, and the dose regime involves repeating the initial oral dose 14 days after the first treatment, then at monthly intervals (Lees *et al*. 2015). Two clinical trials examined the clinical efficacy of mavacoxib treatment in 111 and 124 dogs with naturally occurring OA compared to either meloxicam (Walton *et al*. 2014) or carprofen (Payne‐Johnson *et al*. 2015), respectively. Both studies found mavacoxib to be non‐inferior to the control NSAID over the study periods of 12 weeks and 134 days with a similar rate of adverse events. Due to its prolonged half‐life, however, there is a potential for increased risk of inadvertent overdose by owners, and an inability to cease daily dosing if unwanted side effects do occur (European Medicines Agency 2008).

**Table 1:** A summary of studies involving pharmaceutical treatments of canine osteoarthritis published from 2008 to 2021

Class of drug	Drug	Clinical trials							
		Author	Type of study	Comparison/control group	Study size (number of dogs)	Study period	Outcomes measured	Findings	Level of evidence (Aragon & Budsberg 2005)
NSAIDs	Robenacoxib	Reymond *et al*. (2012)	Multi‐centre, prospective, randomised, blinded, positive‐controlled non‐inferiority clinical trial Clinical cases of dogs with OA	Carprofen	188	12 weeks	Subjective (veterinary and owner assessments) Clinical pathology	Non‐inferiority in efficacy and tolerability of robenacoxib compared to carprofen	II
	Mavacoxib	Walton *et al*. (2014)	Randomised, positive‐controlled non‐inferiority clinical trial Clinical cases of dogs with OA	Meloxicam	111	12 weeks	CMIs: LOAD, HCPI, CBPI Objective (force plate analysis)	Similar clinical efficacy and adverse event rates	II
		Payne‐Johnson *et al*. (2015)	Multi‐centre, randomised, blinded, positive‐controlled non‐inferiority clinical trial Clinical cases of dogs with OA	Carprofen	124	134 days	Subjective owner and veterinary assessment Clinical pathology	Mavacoxib non‐inferior to carprofen, similar safety profile	II
	Enflicoxib	Salichs *et al*. (2021)	Multi‐centre, randomised, blinded, placebo‐controlled clinical trial Clinical cases of dogs with OA	Mavacoxib Placebo	242	6 weeks	Subjective veterinary assessments CMI: CBPI	Both enflicoxib and mavacoxib groups showed significant improvement compared to placebo. Enflicoxib group had higher success rates	II
Piprants	Grapiprant	Rausch‐Derra *et al*. (2016)	Multi‐centre, prospective, randomised, blinded, placebo‐controlled clinical trial Clinical cases of dogs with OA	Placebo	265	28 days	Subjective veterinary and owner assessments CMI: CBPI Clinical pathology	Grapiprant significantly more effective than placebo	II
		de Salazar Alcalá *et al*. (2019)	Randomised, blinded, two‐way crossover study Experimentally induced OA	Firocoxib Untreated control group	18	24 hours	Objective (force plate analysis)	Firocoxib superior to grapiprant. Grapiprant not statistically different from untreated control	IV
		Budsberg *et al*. (2019)	Blinded, three‐way crossover study Experimentally induced OA	Carprofen L‐766	5	48 hours	Subjective (clinical assessment) Objective (force plate analysis)	Carprofen superior to both grapiprant and L‐766	IV
Anti‐NGF mAbs	Ranevetmab	Lascelles *et al*. (2015)	Randomised, double‐blinded, placebo controlled, proof of principle clinical pilot study Clinical cases of dogs with OA	Placebo	26	28 days	CMI: CSOM, CBPI, LOAD Objective (force plate analysis)	Significant improvement with anti‐NGF mAb treatment compared to placebo	II
		Webster *et al*. (2014)	Blinded, placebo‐controlled clinical trial Clinical cases of dogs with OA	Placebo	11	6 weeks	CMI: CBPI	Anti‐NGF mAbs reduced CBPI scores for 4 weeks after treatment	II
	Bedinvetmab	Corral *et al*. (2021)	Double‐blinded, randomised, placebo controlled, multi‐centre clinical trial Clinical cases of dogs with OA	Placebo	287 (comparative phase) 89 (continuation phase)	3‐month comparative phase followed by 6 months of continuation phase	CMI: CBPI	Improvement with bedinvetmab compared to placebo in all assessed time points throughout 3‐month comparative phase Sustained efficacy demonstrated throughout 6‐month continuation phase	II
Opioid	Tramadol	Budsberg *et al*. (2018)	Randomised, blinded, placebo‐controlled crossover study Clinical cases of dogs with OA	Carprofen Placebo	40	10 days	CMI: CBPI Objective (force plate analysis)	Significant improvement with carprofen. No significant difference between tramadol and placebo	II
		Malek *et al*. (2012)	Randomised, prospective, double‐blinded, placebo‐controlled clinical trial Clinical cases of dogs OA	Carprofen ABT‐116 Placebo	49	2 weeks	Subjective veterinary assessment CMI: CBPI Objective (force‐plate analysis, accelerometer activity monitor)	Carprofen or tramadol more effective than ABT‐116 or placebo	II
		Monteiro *et al*. (2019)	Randomised, double‐blinded clinical trial Clinical cases of dogs with OA, and 5 healthy dogs as control group	Reduced dose ketoprofen alone or with tramadol Full dose ketoprofen	25	28 days	CMI: SVAPS Clinical pathology Gastrointestinal endoscopy	Reduced dose ketoprofen and tramadol given in conjunction superior to reduced dose ketoprofen alone	II
Gabapentinoids	Gabapentin	Miles *et al*. (2020)	Randomised, observer‐blinded, crossover study Clinical cases of dogs with OA	Unspecified NSAID + Gabapentin *versus* Unspecified NSAID + tramadol	24	28 days	Objective (force plate analysis) Clinical pathology	Improvement with both tramadol and gabapentin used concurrently with NSAID compared to baseline levels of NSAID alone. Gabapentin plus NSAID more effective than tramadol plus NSAID	II
NMDA receptor antagonists	Amantadine	Lascelles *et al*. (2008)	Randomised, blinded, placebo‐controlled study Clinical cases of dogs with OA	Meloxicam plus placebo Meloxicam plus amantadine	31	6 weeks	CMI: CSOM	Meloxicam plus amantadine more effective than meloxicam plus placebo at reducing clinical signs of OA	II
Cannabinoid	Cannabidiol (CBD oil)	Gamble *et al*. (2018)	Randomised, placebo‐controlled, blinded, crossover study Clinical cases of dogs with OA	Placebo	22	4 weeks	Subjective (veterinary assessment, activity scores) CMI: CBPI Clinical pathology	Decrease pain, increased activity with CBD oil	II
		Verrico *et al*. (2020)	Randomised, double‐blind, placebo‐controlled study Clinical cases of dogs with OA	Placebo High‐dose CBD Low‐dose CBD Liposomal CBD	20	4 weeks	Subjective (veterinary and owner assessments) CMI: HCPI Clinical pathology	Decreased pain with CBD oil treatment	II
		Kogan *et al*. (2020)	Pilot open clinical study Clinical cases of dogs with OA	None	32	90 days	Subjective (veterinary and owner assessments) CMI: CODI Clinical pathology	Improvement in clinical signs of OA with CBD treatment. Of 23 dogs, 21 receiving concurrent gabapentin were comfortable reducing or stopping gabapentin	III
		Brioschi *et al*. (2020)	Randomised clinical study Clinical cases of dogs with OA	NSAID plus gabapentin plus amitriptyline with or without CBD oil	21	12 weeks	CMI: CBPI	Improved CBPI scores at certain time points in CBD‐treated group	II
		Mejia *et al*. (2021)	Randomised placebo‐controlled, double‐blinded, crossover study Clinical cases of canine OA	Placebo	23	6 weeks	CMI: LOAD, CBPI Objective: pressure gait analysis, accelerometery	No significant difference between CBD group and placebo group	II
Corticosteroids	Triamcinolone hexacetonide	Alves et al. (2021)	Randomised, double‐blinded, placebo‐controlled clinical trial Clinical cases of canine OA	Placebo (IA saline)	40 (20 per group)	180 days	CMI: LOAD, CBPI, HVAS, COI Objective: force plate analysis, pedometer, radiography, digital thermography	Significant improvement in treated hips up to 90 days follow up after one IA injection compared to placebo	II
Candidate Structure Modifying OA Drugs	Intra‐articular hyaluronic acid	Carapeba *et al*. (2016)	Randomised, double‐blinded, placebo‐controlled clinical trial Clinical cases of canine OA	IA saline and carprofen and nutraceutical	16 (8 per group)	90 days	Subjective: veterinary assessment CMI: HCPS, CBPI	Improvement in outcomes in both groups over 90 days. IA HA group lower scores in some time points compared to control	II
Regenerative therapies	Autologous MSCs	Cuervo *et al*. (2014)	Multi‐centre, randomised, blinded, parallel group trial Clinical cases of canine hip OA	PRGF	39 (19 ADSC, 20 PRGF)	6 months	Subjective: VAS, joint mobility Objective: radiography	Both ADSCs and PRGF reduced pain and improved function	II
		Vilar *et al*. (2014)	Before and after double‐blinded clinical study Clinical cases of dogs with hip OA	ADSCs alone or with PRGF Non‐treated control	14 (9 treated, 5 healthy control)	6 months	Objective: force plate analysis	ADSC therapy led to significant improvement of lameness for 30 days	III
		Mohoric *et al*. (2016)	Blinded, placebo controlled, clinical trial Clinical cases of bilateral canine stifle OA	IA saline	10 dogs (20 joints)	1 year	Subjective owner assessments Radiographs Synovial fluid assessment	Nine of 10 dogs showed significant improvement up to 1 year after treatment No change in radiographic joint appearance	II
		Vilar *et al*. (2016)	Before and after double‐blinded clinical study Clinical cases of dogs with hip OA	ADSCs alone or with PRGF Non‐treated control	14 (9 treated, 5 healthy control)	6 months	Subjective: VAS, Bioarth assessment scale Objective: force plate analysis	Improvement in force plate analysis in ADSC group with or without PRGF at 30 days after treatment, but not at 90 days. Improved subjective measurements for up to 6 months	III
		Srzentić Dražilov *et al*. (2018)	Before and after clinical study Clinical cases of dogs with hip OA	None	10	90 days (5 followed for 1 to 4 years)	Subjective: veterinary assessment	Improvement with treatment compared to baseline over 90‐day period	III
	Allogenic MSCs	Marx *et al*. (2014)	Before and after pilot clinical study Clinical cases of canine hip OA	SVF	9 (5 ADSC, 4 SVF)	30 days	Subjective: veterinary assessment	All SVF dogs and four of five ADSC dogs showed improvement	III
		Harman *et al*. (2016)	Prospective randomised, blinded, placebo‐controlled efficacy study Clinical cases of dogs with hip OA	Saline placebo	74 (38 treated, 36 control)	60 days	Subjective: veterinary assessments CMI: CSOM	Improved outcomes with ADSC treatment compared to placebo	II
		Kriston‐Pál *et al*. (2017)	Before and after uncontrolled clinical study Clinical cases of elbow OA	None	30 (39 joints)	1 year	Subjective owner and veterinary assessments Arthroscopic and histology exams	Thirty of 39 joints had reported clinical improvements for up to 1 year Regenerated cartilage on arthroscopic and histology exam 1 year after treatment	III
		Shah *et al*. (2018)	Before and after uncontrolled clinical study Clinical cases of dogs with OA in various joints	None	203	10 weeks	Subjective: veterinary assessment	Significant improvement with MSC treatment. IA treatment better outcome than iv treatment. Better outcomes in dogs less than 9 years old	III
		Cabon *et al*. (2019)	Before and after, uncontrolled, open‐labelled clinical study Clinical cases of dogs with OA in various joints	None	22	2 years	Subjective: veterinary and owner assessments	Significant improvement up to 6 months. Eight dogs received second IA injection and showed clinical improvements up to 1 year	III
	PRP	Upchurch *et al*. (2016)	Randomised, prospective, double‐blind, placebo‐controlled trial Clinical cases of dogs with OA	SVF and PRP Saline placebo	22 (12 control, 10 treated)	24 weeks	Subjective: veterinary assessment, VAS CMI: CBPI Objective: force plate analysis	Improvement in treated groups at some time points compared to placebo	II
		Cuervo *et al*. (2020)	Before and after study Clinical cases of dogs with OA	PRP with or without physical therapy	24 (12 PRP alone, 12 PRP + physical therapy)	180 days	Objective: force plate analysis	Improvement with PRP, with or without physical therapy but improvement sustained for longer with physical therapy	III
		Venator *et al*. (2020)	Before and after study. Clinical cases of dogs with OA	None	12	3 months	Objective: force plate analysis	PRP injection improved kinetics for at least 4 weeks, and up to 12 weeks	III
		Alves et al. (2021)	Double blinded, negatively controlled, randomised clinical trial Clinical cases of dogs with OA	Saline IA injection	20 (10 per group)	180 days	CMI: CBPI, LOAD, COI, HVAS	PRP IA injection lead to improvement in outcomes compared to control	II
Gene therapy	IL‐10 plasmid DNA therapy	Watkins *et al*. (2020)	Prospective, randomised, double‐blind, placebo‐controlled study Clinical cases of dogs with OA	Placebo (vehicle)	14 (10 treated, 4 placebo)	8 weeks	Subjective: VAS, veterinary and owner assessments	Treatment well tolerated, trends towards significant decreases in pain	II

NSAID Non‐steroidal anti‐inflammatory drugs, OA Osteoarthritis, CMI Clinical metrology instruments, LOAD Liverpool osteoarthritis in dogs scale, HCPI Helsinki chronic pain index, CBPI Canine brief pain inventory, NGF Nerve growth factor, CSOM Client‐specific outcome measures, SVAPS Standardised veterinarian arthritis pain scale, NMDA N‐methyl d‐aspartate, CBD Cannabidiol, CODI Cincinnati Orthopaedic Disability Index, IA Intra‐articular, HVAS Hudson Visual Analogue Scale, COI Canine Orthopaedic Index, HA Hyaluronic acid, MSC Mesenchymal stem cell, PRGF Plasma rich in growth factors, ADSC Adipose‐derived stem cells, VAS Visual analogue score, SVF Stromal vascular fraction, PRP Platelet‐rich plasma, IL‐10 Interleukin 10

All NSAIDs have potential side effects, the most common of which include gastrointestinal signs such as vomiting, diarrhoea and inappetence, with severe side effects, including gastrointestinal ulceration and renal toxicity, being noted on datasheets of medications to happen rarely to very rarely. The true overall incidence of adverse effects, and whether there is a significant difference between licensed NSAIDs in terms of safety, is unknown (Monteiro‐Steagall *et al*. 2013, Hunt *et al*. 2015).

In summary, NSAIDs provide analgesia for dogs with OA pain, and although there are many licensed for use in dogs with OA, no one licensed NSAID has been shown to be consistently superior to another in terms of efficacy or safety.

### Piprants

#### Grapiprant

Recently, a novel class of non‐steroidal, non‐COX inhibiting drugs, the piprants, have been pursued as treatment options for the management of canine OA. This has led to the development of a prostaglandin E_2_ (PGE_2_) EP4 receptor antagonist (PRA), grapiprant, as a licensed treatment for dogs with mild to moderate OA pain (Galliprant; Elanco).

EP4 is a receptor through which PGE_2_, a key mediator of inflammation and pain, exerts its effects (Nakao *et al*. 2007). By antagonising EP4, grapiprant blocks PGE_2_ mediated sensitisation of sensory neurons and PGE_2_ mediated inflammation, thus producing anti‐inflammatory and analgesic effects without inhibiting the production of prostaglandins as a whole (Lin *et al*. 2006) (Fig 2). Therefore, grapiprant could *theoretically* reduce the risk of side effects caused by the inhibition of COX enzymes (Kirkby Shaw *et al*. 2016).

Only one peer‐reviewed clinical trial involving grapiprant use in dogs for the treatment of naturally occurring OA pain has been published to date. This prospective, randomised, blinded, placebo‐controlled study involving 265 client‐owned dogs with OA confirmed by radiography in at least one appendicular joint examined the clinical effects of grapiprant (2 mg/kg once a day orally) compared to a placebo over the course of 28 days treatment (Rausch‐Derra *et al*. 2016). Outcomes were measured using a clinical metrology instrument (CMI), the canine brief pain inventory (CBPI) (Brown *et al*. 2008) and veterinary assessments, with safety measured by physical examination, clinical pathology results and owner observations. Grapiprant was generally well tolerated, and it was found to have improved pain scores compared to placebo, with a treatment success rate of 48.1% for grapiprant treated dogs compared to 31.3% for dogs receiving a placebo (P=0.0315). These measurements of treatment success were based on a previous definition by Brown *et al*. (2013a), using the CBPI and an overall impression score of the same or better on day 28 compared to day 0. Studies comparing the analgesic efficacy of grapiprant to NSAIDs have so far only examined acute pain control for the 24 to 48‐hour period following experimental induction of arthritis or synovitis. Both studies found the NSAID (firocoxib or carprofen) to have superior analgesic properties in that timeframe (Budsberg *et al*. 2019, De Salazar Alcalá *et al*. 2019) (Table 1). However, it should be noted that this is a very short timeframe for treatment and is in an acute pain experimental model of induced OA rather that a clinical setting. Further studies comparing the efficacy of grapiprant to that of NSAIDs would be of use in improving the evidence behind its use and as a first‐line treatment for OA pain in dogs.

Potential side effects of grapiprant include vomiting [very common (more than one in 10 animals treated)], diarrhoea and inappetence [common (between one and 10 animals in 100 animals treated)], with these side effects being generally mild and transient. In very rare cases (less than one in 10,000 animals treated), haemorrhagic diarrhoea or haematemesis has been reported (National Office of Animal Health 2021b). The recommended daily dose of grapiprant is 2 mg/kg once a day. In a study examining the safety of daily grapiprant administration at doses up to 50 mg/kg orally once a day for 9 months in healthy Beagles in an experimental setting, it was well tolerated with no renal or hepatic toxicity noted suggesting the safety of long‐term oral administration of grapiprant to dogs (Rausch‐Derra *et al*. 2015).

Piprants are a new class of drugs that offer a more targeted mechanism of action than COX‐inhibiting NSAIDs (Kirkby Shaw *et al*. 2016). However, their clinical efficacy in the long‐term treatment of canine OA compared to NSAIDs is, as yet, unknown, and larger clinical trials comparing these drugs would improve the evidence.

### Paracetamol and paracetamol/codeine

Paracetamol (acetaminophen) is an analgesic and antipyretic, and is a common first‐line treatment for OA pain in humans (Onakpoya 2020). It has a complex mechanism of action, which is still not completely understood, functioning as both an inhibitor of COX peripherally and centrally, as well as acting on other central antinociception pathways such as serotonergic pathways, the endocannabinoid system and the l‐arginine/NO pathway (Przybyła *et al*. 2021). Paracetamol on its own is not licensed for use in dogs in the UK, but preparation of paracetamol and codeine phosphate (400 mg paracetamol/9 mg codeine phosphate) is licensed in the UK for the treatment of acute pain of traumatic origin, as a complementary treatment in pain associated with other conditions, and for postoperative analgesia (Pardale‐V, Dechra Veterinary Products). This is licensed for a treatment duration of up to 5 days. Codeine, an opioid, has a low oral bioavailability in dogs and it is unknown whether it effectively contributes to the analgesic effects of this product (Kukanich 2010).

In humans, recent Osteoarthritis Research Society International treatment guidelines no longer recommend the use of paracetamol as a single‐agent in the treatment of knee, hip and polyarticular OA (Bannuru *et al*. 2019). Previously, the use of paracetamol had been advised as a first‐line treatment, but evidence in recent meta‐analyses of human trials show it to have little to no efficacy in the treatment of OA in humans (Machado *et al*. 2015, Da Costa *et al*. 2017, Bannuru *et al*. 2019). In canines, there are no published studies examining the analgesic efficacy of paracetamol alone, in combination with codeine, or as an adjunctive analgesic with other medications such as NSAIDs in dogs with chronic OA pain. This is a gap in the current evidence base. In practice, paracetamol can be used (off licence) concurrently with an NSAID as an adjunctive analgesia, or as an alternative to an NSAID in those dogs where NSAID use is not tolerated (Pettitt & German 2015).

There is no published data on the analgesic efficacy of paracetamol in canine OA specifically, and use of Pardale‐V (Dechra) of more than 5 days duration, or concurrently with an NSAID, is off licence. This highlights a gap in the current evidence base of pharmaceutical treatments for canine OA.

### Antinerve growth factor monoclonal antibodies

Nerve growth factor (NGF) is a soluble signalling protein, released from peripheral tissues in response to noxious stimuli. It has an important role in nociceptor sensitisation in both acute and chronic pain states, including OA, by increasing peripheral sensitisation through phenotypic alterations, increasing the expression of pro‐nociceptive neurotransmitters and inducing inflammatory mediator release in the periphery (Enomoto *et al*. 2019).

#### Bedinvetmab

In 2020, a canine‐specific anti‐NGF mAb product, bedinvetmab (Librela; Zoetis), received approval from the European Medicines Agency. This product is licensed in the UK as a once monthly subcutaneous injection for dogs over 12 months of age, at a dose of 0.5 to 1.0 mg/kg.

In a double‐blinded, placebo‐controlled, multi‐centre, randomised controlled trial, Corral *et al*. (2021) investigated the efficacy of monthly bedinvetmab injections in 287 dogs with OA. The study had a 3‐month comparative phase, where CBPI scores were used as primary outcomes in one group treated with bedinvetmab, compared to another group given a placebo. Improved CBPI scores where observed in all assessed time points in this trial in the bedinvetmab‐treated groups compared to placebo. The study then had a continuation phase, involving 89 dogs that showed clinical improvement with bedinvetmab treatment continuing with monthly injections of bedinvetmab for a further 6 months. Sustained efficacy was shown over this timeframe in terms of CBPI scores, although the study lacked objective outcome measurements such as force plate analysis.

Concurrent use of anti‐NGF mAbs with NSAIDs in humans in clinical trials has shown a rapid progression of OA (Hefti 2020). The concurrent use of bedinvetmab with an NSAID (carprofen) has only been investigated in young, healthy laboratory dogs without OA for a period of 2 weeks (Krautmann *et al*. 2021), and the long‐term concurrent use of these medications in dogs with OA has not been investigated. Therefore, it cannot be currently recommended to administer bedinvetmab and an NSAID concurrently. The only noted adverse reaction on the datasheet for Librela (Zoetis) is an uncommon mild reaction at the injection site (National Office of Animal Health 2021c).

Initial evidence for the effectiveness of anti‐NGF mAbs shows promise in providing an alternative treatment option for canine OA (Webster *et al*. 2014, Lascelles *et al*. 2015, Corral *et al*. 2021), with bedinvetmab licensed for the alleviation of pain associated with OA in dogs.

### Opioids

#### Tramadol

Tramadol has been used as an analgesic in humans since the 1970s (Schenck & Arend 1978) and exerts its analgesic effects via several mechanisms. It is a mu‐opioid agonist and a serotonin and noradrenaline reuptake inhibitor (Grond & Sablotzki 2004). The effects on the mu‐opioid receptor are predominantly due to the tramadol metabolites, especially the *O*‐desmethyltramadol (M1) metabolite. Some dogs (and it is unknown what proportion) are unable to produce this metabolite, hence reducing the mu‐opioid analgesic effects in some canines (Kukanich & Papich 2004, Perez Jimenez *et al*. 2016).

Since 2018, there are now two licensed tramadol hydrochloride products available for use in the reduction of acute and chronic mild soft tissue and musculoskeletal pain in dogs in the UK (Tralieve; Dechra and Tramvetol; Virbac).

The evidence in the literature supporting the use of tramadol as an analgesic in the treatment of canine OA describes mixed results. Budsberg *et al*. (2018) compared subjective and objective outcome measurements in 40 dogs with OA when treated with either carprofen, tramadol or a placebo for 10 days. A significant improvement in all outcome measures was found with carprofen treatment, but not with placebo or tramadol. It was concluded in this study that tramadol provided no clinical benefit in the treatment of canine OA; however, the time period of the study was short, and the number of dogs in each treatment group was small. Malek *et al*. (2012), however, found an improvement in owner assessed mobility scores in dogs treated with either tramadol or carprofen compared to ABT‐116 [a transient receptor potential vanilloid 1 (TRPV1) antagonist] or placebo. However, no difference was found between groups on objective kinetic gait analysis. This study involved 49 dogs with OA treated for 2 weeks.

Two studies have examined concurrent use of tramadol with an NSAID compared to treatment with an NSAID alone (Monteiro *et al*. 2019, Miles *et al*. 2020). Monteiro *et al*. (2019) found an increased analgesic efficacy in 20 dogs when tramadol was used in conjunction with reduced dose ketoprofen over 28 days. Miles *et al*. (2020) also found an improvement in objective measurements of lameness such as force plate gait analysis in a group of 18 dogs receiving both tramadol and an NSAID for 28 days compared with NSAID as a sole agent.

With a limited number of clinical studies investigating the efficacy of tramadol for the treatment of canine OA pain, and the known individual variability of the production of the M1 metabolite in dogs (Kukanich & Papich 2004), it is difficult to draw firm conclusions over its clinical effectiveness. Budsberg *et al*. (2018) suggested that when tramadol was used as a sole analgesic for periods of less than 2 weeks, it provided insufficient analgesia for OA pain. However, there may be a beneficial role when used in conjunction with other analgesics as part of a multi‐modal approach to pain management in some dogs (Monteiro *et al*. 2019).

### Gabapentinoids

#### Gabapentin and pregabalin

Gabapentin is a synthetic analogue of γ‐aminobutyric acid (GABA) and is used in human medicine as an anti‐epileptic drug, and in the treatment of chronic neuropathic pain and fibromyalgia (Calandre *et al*. 2016). Its mechanism of action is not fully understood, but it is believed to exert its main effects by selectively inhibiting voltage‐gated calcium channels containing the alpha2delta‐1 subunit, leading to reduced neurotransmitter release and lessening of postsynaptic excitability (Sills 2006). Pregabalin has a similar mechanism of action to gabapentin, but it has a longer half‐life and higher oral bioavailability (Salazar *et al*. 2009).

Previous pharmacokinetic studies have suggested a dosage of gabapentin in dogs of 10 to 20 mg/kg orally every 8 hours (Kukanich & Cohen 2011) compared to a dosage of pregabalin of 4 mg/kg every 12 hours to achieve the therapeutic plasma concentration seen in humans (Salazar *et al*. 2009). The therapeutic plasma concentrations in dogs are unknown and there are currently no licensed products of either gabapentin or pregabalin available for veterinary use in the UK.

There are a limited number of published studies involving the use of gabapentin and pregabalin in dogs, and only one published abstract describing a study examining the use of gabapentin as an adjunctive treatment with NSAIDs in canine OA (Miles *et al*. 2020). This study involved objective measurements of gait analysis of a small cohort of 24 dogs with OA, receiving either tramadol or gabapentin for 4 weeks in addition to an NSAID. Both tramadol and gabapentin led to an improvement in weight bearing (Miles *et al*. 2020).

The evidence for the use of gabapentinoids in canine OA is currently lacking. More published high‐quality clinical trials are needed to examine the efficacy of gabapentin and pregabalin in the dog, in order to give a greater evidence‐base behind their usage.

### *N*‐methyl d‐aspartate receptor antagonists

#### Amantadine and memantine

Amantadine, first developed as an antiviral medication and also used to treat Parkinson's disease in humans, exerts its analgesic effects by antagonising *N*‐methyl d‐aspartate (NMDA) receptors (Fisher *et al*. 2000). Memantine is also an NMDA receptor antagonist and is a more potent congener of amantadine (Johnson & Kotermanski 2006). Although neither drug is licensed in dogs, suggested doses of amantadine are 3.0 to 5.0 mg/kg orally once a day. A study examining the use of memantine in the treatment of compulsive disorders in dogs suggested doses of memantine of 0.3 to 0.5 mg/kg orally twice a day initially, increasing to 1.0 mg/kg twice a day if necessary (Schneider *et al*. 2009).

There is only one published study examining the use of amantadine in dogs with OA (Lascelles *et al*. 2008). This study showed an improvement in subjective veterinary assessment of OA pain, and in the client‐specific outcome measure (CSOM) CMI in 31 dogs when amantadine was given in conjunction with meloxicam, compared to meloxicam with placebo.

NMDA antagonists could be beneficial as adjunctive analgesics in managing chronic OA pain (Lascelles *et al*. 2008); however, this was found in one small study, and larger clinical trials are required to improve the evidence behind their use. There are no published clinical trials involving the use of memantine in dogs with OA and both the use of amantadine and memantine are off licence in the UK.

### Cannabinoids

#### Cannabidiol (CBD oil)

Cannabinoids have gained attention in recent years for their potential efficacy as analgesics in patients with chronic pain. They have a complex mechanism of action, acting on peripheral, spinal and supra‐spinal sites to exert antinociceptive and antihyperalgesic effects (Richardson 2000, Landa *et al*. 2016).

Clinical trials investigating the use of CBD oil in dogs with OA have had mixed results, and are all of low sample size, involving up to a total of 32 dogs (Table 1). Two randomised crossover studies comparing CBD oil treatment to placebo found a beneficial effect in reduction of clinical signs by subjective measurements and validated CMIs over study periods of 4 weeks in 20 and 22 dogs, respectively (Gamble *et al*. 2018, Verrico *et al*. 2020). However, in another randomised, double‐blinded, crossover study involving 23 dogs, Mejia *et al*. (2021) found no significant difference between CBD oil treatment and placebo over the course of 6 weeks, based on CMI outcomes and objective pressure gait analysis. Two other published studies have suggested an improvement in clinical signs of OA in dogs treated with CBD oil as part of a multi‐modal analgesic plan (Brioschi *et al*. 2020), and with gabapentin (Kogan *et al*. 2020). However, both studies have limitations. The latter study had no control group, and did not use validated pain scoring systems to subjectively measure outcomes (Kogan *et al*. 2020), and the former involved dogs already receiving an NSAID, gabapentin and amitriptyline (Brioschi *et al*. 2020). Both also involved small numbers of dogs, with sample sizes of 32 and 23, respectively.

Currently, there is no licence for the use of CBD oil in dogs in the UK, and veterinary surgeons should be aware of the legal position regarding prescribing these medications. A recently published information sheet from the British Small Animal Veterinary Association (BSAVA) outlines the current position in the UK (Wessmann *et al*. 2016). There is currently very limited available evidence for the efficacy of CBD oil for the treatment of canine OA pain.

### Tricyclic antidepressants

#### Amitriptyline

Amitriptyline is a tricyclic antidepressant (TCA) and commonly used as a treatment for neuropathic pain in humans. TCAs exert their analgesic effects by several mechanisms of action, including NMDA and adrenergic receptor antagonism, serotonin and noradrenaline reuptake inhibition, voltage‐gated sodium channel blockade, and enhancement of adenosine and GABA_B_ receptor activity (Dharmshaktu *et al*. 2012).

There are currently no published clinical trials investigating the analgesic efficacy of TCAs in dogs with OA pain, and there is no licensed preparation of amitriptyline for dogs.

### Corticosteroids

Corticosteroids are thought to have an analgesic effect in OA due to their anti‐inflammatory action (Johnston & Budsberg 1997). Their use in OA management is controversial (Behrens *et al*. 1975, Murphy *et al*. 2000), and due to the well‐documented risks of long‐term corticosteroid use they should be used with caution if given long term. Prednoleucotropin, an oral prednisolone and cinchophen combination product has been used for the treatment of OA in dogs (McKellar *et al*. 1991) but is no longer licensed in the UK.

Intra‐articular (IA) injections of long‐acting preparations of corticosteroids (methylprednisolone acetate, triamcinolone acetonide and triamcinolone hexacetonide) in humans with OA have been shown to have a short‐term benefit in alleviation of pain compared to placebo (Najm *et al*. 2021). Methylprednisolone acetate is licensed for IA use in the dog for inflammatory conditions (Depo‐medrone V; Zoetis), therefore its use in OA (typically considered a non‐inflammatory condition, although now understood to have an inflammatory component) under the licence is debatable (Sokolove & Lepus 2013). Clinical trials into its effectiveness and safety in naturally occurring OA are lacking. A recent clinical trial found improvements in weight bearing for up to 90 days in a group of 20 dogs following a single IA injection of an unlicensed preparation of the corticosteroid triamcinolone hexacetonide in OA hip joints compared to a control group receiving a placebo (Alves *et al*. 2021b).

Veterinary practitioners should consider the contraindications for corticosteroids, such a septic arthritis, and safety concerns over potential cartilage damage with long term IA corticosteroids before use (Chunekamrai *et al*. 1989, Farquhar *et al*. 1996, Murphy *et al*. 2000). Long‐term efficacy and safety studies of IA corticosteroid use in clinical trials in dogs are lacking.

### Candidate structure modifying OA drugs

Several drugs have been investigated as potential structure modifying OA drugs (SMOADs). SMOADs are defined as drugs that can delay, stabilise or repair OA lesions in affected joints rather than just alleviating the symptoms of OA (Sevalla *et al*. 2000, Sunaga *et al*. 2012). However, no evidence currently exists that these drugs are able to stabilise or repair OA lesions in vivo. Examples of these medications include pentosan polysulphate (PPS), polysulphated glycosaminoglycans (PSGAGs), hyaluronic acid (HA) and doxycycline. Little new evidence has emerged regarding the use of these treatments in canine OA over the past decade.

Sodium PPS is licensed for treatment for OA in dogs in the UK [Osteopen; Chanelle Pharma, Cartrophen Vet; Arthropharm (Europe) Ltd]. It has a wide range of pharmacological activities, including anticatabolic activities in articular cartilage, anti‐inflammatory actions, increasing hyaluronan production from synoviocytes and thrombolytic activity that could enhance blood supply to affected joints (Ghosh 1999). Studies investigating the efficacy of PPS as a treatment for OA in dogs have shown improvements in outcomes compared to placebo; however, they are limited and not all based upon objective outcomes (Bouck *et al*. 1995, Read *et al*. 1996, Innes *et al*. 2000, Smith *et al*. 2001, Budsberg *et al*. 2007).

The mechanisms by which PSGAGs are believed to be through the inhibition of matrix metalloprotease (MMP) enzymes, therefore having a preventive effect on matrix molecule degradation in articular cartilage (Sevalla *et al*. 2000). PSGAGs were found to have a moderate level of evidence in a previous systematic review based on two included randomised controlled trials, although it was concluded that further studies were needed (Sandersoln *et al*. 2009).

Doxycycline has been investigated as a SMOAD due to in vitro studies suggesting a potential to slow cartilage degeneration (Shlopov *et al*. 1999). However, evidence behind its use as a treatment for canine OA in a clinical setting is limited, and previous systematic reviews have found no evidence for its use in the treatment of canine OA (Aragon *et al*. 2007, Sandersoln *et al*. 2009).

A recent small cohort clinical trial in dogs with naturally occurring OA secondary to hip dysplasia, compared eight dogs treated with IA injections of a low molecular weight HA with a control group receiving saline IA injections plus oral carprofen and a nutraceutical containing glucosamine, chondroitin sulphate and collagen (Carapeba *et al*. 2016) (Table 1). Dogs in both groups showed an improvement from baseline scores when assessed by subjective measurements [CPBI, Helsinki Chronic Pain Scale (HCPS) and veterinary assessments] up to 90 days after treatment. However, a greater improvement was shown in the dogs treated with IA HA. There is currently no licensed preparation of IA HA for use in dogs in the UK, and further evidence behind its use in canine OA is required before conclusions can be drawn as to its effectiveness in clinical cases.

As this review is an update focusing primarily on recent treatments, or updated clinical evidence behind existing treatments, we refer the reader to previous systematic reviews outlining the evidence behind candidate SMOADs (Aragon *et al*. 2007, Sandersoln *et al*. 2009).

#### Mesenchymal stem cells

Mesenchymal stem cells (MSCs) are progenitor cells with the ability to differentiate into numerous cell types, such as cells of connective tissue, bone and cartilage (Caplan 1991). They can be derived from a variety of tissue including adipose tissue and bone marrow, which have both been shown to be a source of MSCs in dogs (Screven *et al*. 2014).

Although MSC treatment is often termed a regenerative therapy, the exact mechanisms by which MSCs exert their effect are still under investigation. It was previously thought that the main mechanism of action of the stem cells was that, once injected into the site of an OA lesion, they undergo differentiation into chondrocytes, therefore repairing the OA lesion (Scharstuhl *et al*. 2007). However, it is now thought that the effects of MSCs are exerted primarily through their secreted factors, including extracellular vesicles (EVs) and bioactive molecules such as chemokines, cytokines and growth factors, termed the secretome (Tofiño‐Vian *et al*. 2018, Villatoro *et al*. 2019). These paracrine factors have a range of immunomodulatory, anti‐inflammatory, angiogenic and anti‐apoptotic properties (Phinney & Pittenger 2017, Mocchi *et al*. 2020). Because of this growing knowledge of the importance of these secreted factors in the mechanism of action of MSCs, it has recently been proposed that they should now be known as “Medicinal Signalling Cells” rather than MSCs (Caplan 2017).

Adipose tissue‐derived MSCs (ADSCs) are the favoured source of MSCs for clinical use in the dog due to the relative ease of accessibility and rapid rate of proliferation in culture (Zhu *et al*. 2008). MSCs can be autologous, allogenic or xenogenic depending on whether the cells are derived from the same dog that will be the recipient of the cells, from a different donor, or from a different species, respectively (Cuervo *et al*. 2014, Cabon *et al*. 2019, Daems *et al*. 2019). In autologous canine ADSC treatment, subcutaneous adipose tissue is collected from the dog that will be the recipient of the ADSCs, and the cells are cultured and expanded in vitro, followed by IA injection into the affected joint (Cuervo *et al*. 2014). The process of culturing the cells in vitro ensures a consistent and enriched MSC population, but can take 7 to 10 days, therefore increasing the cost of treatment (Voga *et al*. 2020). Alternatively, autologous MSC preparations can be performed by minimal manipulation, allowing for a faster preparation of an MSC product patient‐side. These products, such as stromal vascular fraction contain less MSCs per millilitre, and also contain other cell types (Franklin *et al*. 2018).

Clinical trials involving the use of MSCs in clinical cases of dogs with OA are generally of low sample size and involve different methodologies, but have shown some beneficial outcomes. A systematic review of the treatment of naturally occurring hip OA in dogs with ADSCs has been recently published (Olsson *et al*. 2021). This systematic review included six clinical trials, and concluded that there was evidence that the use of ADSCs by IA injection led to an improvement in clinical signs associated with OA in dogs and that both autologous and allogenic ADSCs were well tolerated with no adverse events noted. One of these studies involved comparing 38 dogs with OA treated with IA ADSCs compared to 36 given a saline placebo, and found significant improvement in subjective CSOM in the treated group (Harman *et al*. 2016). Other studies included smaller numbers of dogs with 18 dogs or less. Only two studies utilised objective outcome measurements of lameness with force plate gait analysis (Vilar *et al*. 2014, Vilar *et al*. 2016), with the other four studies using subjective outcome measurements, including owner visual analogue scales (VAS) (Cuervo *et al*. 2014), CSOM (Harman *et al*. 2016, Srzentić Dražilov *et al*. 2018) and veterinary assessments (Cuervo *et al*. 2014, Marx *et al*. 2014, Harman *et al*. 2016, Vilar *et al*. 2016). Clinical trials have also examined the effect of MSC treatment on OA in the stifle joint and elbow joint in the dog (Mohoric *et al*. 2016, Kriston‐Pál *et al*. 2017). A larger study involving cases of OA in various joints in 203 dogs found significant improvements on subjective veterinary assessments of clinical signs of OA 10 weeks after either IA (n=128) or intravenous (iv) (n=65) treatment with an allogenic MSC preparation, although these improvements were more significant after IA than iv treatment (Shah *et al*. 2018). However, there was no control group in this study, and the outcomes were not assessed by a validated CMI.

The length of treatment efficacy after a single IA injection of MSCs differed between studies, and is limited by the length of some studies but has been reported to be from 1 month (Vilar *et al*. 2014) up to 6 months following a single IA injection (Cuervo *et al*. 2014, Cabon *et al*. 2019).

The collection of adipose tissue or bone marrow for autologous MSC preparation involves some risk, with the potential for donor site morbidity to occur, and the need for a general anaesthetic (Redondo *et al*. 2007, Espinel‐Rupérez *et al*. 2019). Allogenic MSC preparations can reduce this risk, but potentially hold a greater risk of an adverse immune response to treatment (Oliveira *et al*. 2017, Cabon *et al*. 2019). The administration of the product, like other IA products, also requires sedation or general anaesthesia (Redondo *et al*. 2007). However, minimal adverse effects to MSC treatment in dogs with OA have been reported in studies to date. These include local injection site reactions (Cabon *et al*. 2019), and a mild skin allergy in one dog (Shah *et al*. 2018).

A future area for development in MSC treatment research lies in examining EVs released from MSCs, as a potential cell‐free version of treatment. EVs contain a number of biologically active signalling molecules that lead to the beneficial effects of MSCs in OA treatment, and by isolating EVs for use in therapies towards OA there may be less of an immune response towards treatment (Bari *et al*. 2019, Li *et al*. 2019). This area of research, however, is still in its infancy.

In veterinary practice, there are currently no defined guidelines for the dose or frequency of MSC treatment in clinical scenarios. Several companies offer assistance in the culture and preparation of MSCs, but there is currently a lack of standardisation of protocols between companies in terms of methods and clinical approach.

Overall, MSCs show potential as an alternative, or adjunctive, treatment option in cases where conventional treatment is not providing adequate outcomes. Commercial systems are available, but there is a need for greater regulation and standardisation of methods. There is also a need for larger, multi‐centre, randomised controlled clinical trials using a standardised clinical approach and method to better evaluate outcomes in clinical cases of canine OA (Sasaki *et al*. 2019, Olsson *et al*. 2021).

#### Platelet‐rich plasma

Platelet‐rich plasma (PRP) is a blood‐derived product, and consists of plasma with a higher concentration of platelets than is present in peripheral blood (Rossi *et al*. 2019). It can also contain varying amounts of leukocytes (Carr *et al*. 2015, Alves *et al*. 2021a). Platelets are integral to blood clotting, and release growth factors such as platelet‐derived growth factor, transforming growth factor beta, epidermal growth factor and vascular endothelial growth factors (Verheul *et al*. 1997, Coppinger *et al*. 2004). These growth factors stimulate processes such as angiogenesis, and chondrocyte proliferation, and reduce processes such as chondrocyte apoptosis (Collins *et al*. 2021).

The clinical use of PRP therapy for canine OA involves drawing a blood sample from the affected dog, which is then processed to produce plasma highly concentrated in platelets (Perego *et al*. 2020). This is then injected into the IA space of the OA affected joint. Different commercial systems, both external and in‐house, for the processing of canine PRP exists, and, like MSCs, there is currently no standardised method of preparation (Carr *et al*. 2015, Franklin *et al*. 2015).

The efficacy of PRP in OA treatment in humans is still under debate, with some systematic reviews on their use in human medicine showing an overall low level of evidence (Gato‐Calvo *et al*. 2019), and others concluding that PRP was more efficacious than other conservative methods of OA treatment (Hong *et al*. 2021). In dogs, there are a limited number of clinical trials published, all involving small numbers of dogs (up to 24 dogs), and different methodologies (Cuervo *et al*. 2020).

Venator *et al*. (2020) investigated objective force plate analysis in dogs with non‐stabilised CCL rupture treated with a single IA PRP injection. The study found an improvement in kinetics for a minimum of 4 weeks after treatment, but there was no control group to act as a comparison. Upchurch *et al*. (2016) also found improvements in objective and subjective outcome measures in dogs with hip OA after IA and iv PRP and MSC treatment compared to placebo in 12 dogs. Another study examined objective lameness outcomes in dogs receiving IA PRP compared to another group receiving IA PRP as well as physiotherapy (Cuervo *et al*. 2020). Both groups improved compared to baseline levels. Five case reports of PRP treatment for refractory OA describe beneficial outcomes, but it is difficult to extrapolate these outcomes to the wider dog population due to the low number of case studies and lack of a control group (Catarino *et al*. 2020). A recent study by Alves *et al*. (2021a) compared outcomes of groups of 20 dogs with naturally occurring hip OA receiving either two injections of PRP given 14 days apart, or two IA saline injections as a negative control. Outcomes were measured by four different validated CMIs [LOAD, CBPI, Canine Orthopaedic Index (COI) and Hudson Visual Analogue Scale (HVAS)], and dogs were assessed at intervals up to 180 days after treatment. A significant improvement was found in the PRP group compared to control, lasting for approximately 130 days.

No severe adverse reactions have been reported in the literature to date, although studies have described adverse reactions related to transient pain at the injection site which usually improves within 48 to 72 hours (Alves *et al*. 2021a). The need for sedation or general anaesthetic to administer the IA treatment also holds some level of risk (Redondo *et al*. 2007).

Overall, PRP treatment requires further standardisation and regulation of different methods (Carr *et al*. 2015). Larger scale, multi‐centre, randomised controlled clinical trials are required before firm conclusions over its effectiveness as a treatment for canine OA in practice can be drawn.

### Gene therapy

#### Anti‐inflammatory cytokine plasmid DNA therapy

Anti‐inflammatory cytokines, such as interleukin (IL)‐10 can inhibit the production of pro‐inflammatory cytokines, such as IL‐6, IL‐1β and tumour necrosis factor α, as well as downregulating MMP production and preventing chondrocyte apoptosis (John *et al*. 2007, Kapoor *et al*. 2011). Previous studies investigating the use of IL‐10 as an OA therapy have found limited benefits due to the short half‐life in vivo (Chernoff *et al*. 1995). Recent investigations into novel therapies for OA have included research into the use of gene therapies that encode for anti‐inflammatory cytokines, such as IL‐10. In a recent randomised double‐blinded placebo‐controlled clinical trial in 14 dogs with naturally occurring OA, IA targeted IL‐10 plasmid DNA therapy demonstrated some beneficial effects on reducing clinical signs of OA based on owner and veterinary subjective VAS. The same paper described safety studies, and concluded that the treatment was well tolerated (Watkins *et al*. 2020).

More work is required before IL‐10 plasmid DNA therapies are available commercially, but this is an area for future development in OA treatment (Schulze‐Tanzil 2021).

### Conclusion

A greater understanding of the biology and pain mechanisms of OA has led to a growing number of pharmaceutical treatment options for canine OA in the past decade. With the advent of novel medications such as anti‐NGF mAbs and piprants, as well as a growing number of adjunctive analgesics, and greater availability of regenerative therapies in veterinary medicine, veterinary practitioners now have more therapeutics to offer, hopefully improving the welfare of dogs with this condition. Although a search for a curative treatment of OA is ongoing and, as yet, elusive, there are still exciting areas for future development, such as gene and mRNA therapy (Schulze‐Tanzil 2021).

This review highlights that there is a need for larger scale, randomised controlled clinical trials to improve the evidence underpinning treatments to ensure that both veterinary professionals and animal caregivers can have more confidence in the effectiveness of treatments in clinical cases. It should also be noted that a multi‐modal approach to treatment, incorporating not only different types of pharmaceuticals but also weight management, nutraceuticals, physiotherapy and other complementary therapies is important in the non‐surgical treatment of this whole joint disease (Mlacnik *et al*. 2006).

#### Conflict of interest

None of the authors of this article has a financial or personal relationship with other people or organisations that could inappropriately influence or bias the content of the paper.
